# Supplementary material for: Histopathological profile of cervical punch biopsies and risk factors associated with high-grade cervical precancerous lesions and cancer in northwest Ethiopia
Source: PLoS One. 2022 Sep 12;17(9):e0274466. doi: 10.1371/journal.pone.0274466 (PMC9467373; doi:10.1371/journal.pone.0274466)
Supplement: Attachment — Submitted filename: PLOS ONE REVIEWERS COMMENT.docx [file pone.0274466.s001.docx]

REVIEWER’S COMMENTS

**Histopathological profile of cervical punch biopsies and risk factors associated with high-grade cervical precancerous lesions and cancer in northwest Ethiopia**

The paper aimed to determine the histopathological profile of 335 women and risk factors associated with high-grade cervical lesions and cancer over a period of 2.5 years in northwest Ethiopia. It is necessary to do this type of work, to publicize the cervical characteristics of the patients. As well as unifying criteria between pathologists.

The paper is interesting and fairly well written. The data which are presented are moderate to clearly presented though there are several issues that deserve more attention or should be improved.

Specific points: **Methods**

1. The authors should give some more information on how current screening in Ethiopia or area of study is organized. Are cervical scrapes performed? Is there any way of screening with pap-smears after which women are referred for colposcopy with cervical biopsy? This will give the reader a better understanding why cervical punch biopsies were taken blindly in the current study.
2. This being a prospective study, authors should explain how sample size was determined? The authors should clearly explain how they reached at a sample size of 335.

**Results**

1. It is good to note that Table 1 has both demographic and clinical characteristics. What does Bahir Dar 2021 on line number 197 mean? Unless it is in agreement with this journal requirement I recommend that the authors delete it from all the tables, because it does not make sense.

- In Table 1
  - you report 335 participants but the sum of life time number of sexual partners is (163 + 169=332), number of deliveries is (59+86+197=342) exceeding number of participants in the study.
  - It is also indicated that there were 70 participants who reported to have used hormonal contraceptives but the breakdown for years of hormonal contraceptive use is (32+15+10 =57).
  - The women who had heard about cervical cancer are 142 but when they were asked how it is transmitted only (10+114=124) responded. Some are missing
  - The same applies to those screened for cervical cancer in the last 5 years and high-risk Human papillomavirus (HR-HPV) detection

**Conclusion**

2. Even though the majority of the study participants lived in the rural setting (66%), had no formal education (77.3%) and never heard about cervical cancer (57.6%), the conclusion does not specifically address this population.

**Minor issues**

1. Authors should proof read the manuscript because there are a few typos and language that need to be corrected. For example in results section line number 174, in discussion section line number 240, 245, 324 amongst others.
2. Authors also need to check that references, words, symbols and stats are not running into each other. For example words vs references are in background section line number 72, discussion section line number 235 etc
